# Overcoming Barriers to Axonal Regeneration in Spinal Cord Injury: Mechanistic Insights and Therapeutic Frontiers

**DOI:** 10.1002/cns.71034

**Published:** 2026-07-21

**Authors:** Jiaxin Gao, Tian Li, Qingping Su, Cai Jiang, Zhonghua Lin

**Affiliations:** ^1^ Rehabilitation Medicine Center Fuzhou University Affiliated Provincial Hospital Fuzhou China; ^2^ Department of Radiology Fuzhou University Affiliated Provincial Hospital Fuzhou China; ^3^ Department of Complementary Medicine University of Johannesburg Johannesburg South Africa

**Keywords:** axonal regeneration, repair, spinal cord injury

## Abstract

**Background:**

Spinal cord injury (SCI) is a devastating central nervous system disorder that often causes permanent neurological deficits, while effective disease‐modifying therapies remain lacking. Functional recovery depends on axonal regeneration, which is limited by the inhibitory post‐injury microenvironment and the reduced regenerative capacity of mature neurons.

**Methods:**

This review summarizes the mechanisms underlying axonal regeneration failure after SCI and discusses recent advances in regenerative strategies and their translational progress.

**Results:**

Current evidence indicates that impaired axonal regeneration results from both extrinsic inhibitory factors and intrinsic neuronal growth limitations. Regenerative strategies, including modulation of glial scar dynamics, degradation of inhibitory extracellular matrix components, reconstruction of a permissive regenerative microenvironment, stem cell transplantation, biomaterial scaffolds, activation of intrinsic growth programs, and neuromodulation, have shown promising effects in preclinical studies. However, clinical translation remains limited by challenges related to therapeutic timing, delivery efficiency, biosafety, scalability, and insufficient clinical evidence.

**Conclusions:**

Future SCI repair will likely require combinatorial strategies targeting multiple regenerative mechanisms while improving clinical feasibility. This review integrates current mechanistic and translational advances to support the development of more effective regenerative therapies for SCI.

## Graphical abstract

This review summarizes the key intrinsic and extrinsic barriers to axonal regeneration after spinal cord injury and outlines current strategies to rebuild a growth‐permissive microenvironment and enhance neuronal regenerative capacity.

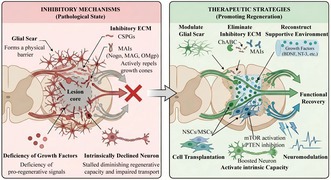

**Abbreviations**
3D: three‐dimensional
AAV: adeno‐associated virus
AMPK: AMP‐activated protein kinase
BDNF: brain‐derived neurotrophic factor
B‐RAF: B‐RAF proto‐oncogene, serine/threonine kinase
ChABC: chondroitinase ABC
CNS: central nervous system
CSPGs: chondroitin sulfate proteoglycans
DTMS: double‐target magnetic stimulation
ECM: extracellular matrix
ELF‐MF: extremely low‐frequency magnetic fields
GAP‐43: growth‐associated protein 43
GDNF: glial cell line‐derived neurotrophic factor
HGF: hepatocyte growth factor
IGF‐1: insulin‐like growth factor‐1
KLFs: Krueppel‐like factors
KO: knockout
LAR: leukocyte common antigen‐related receptor
LV: lentivirus
MAG: myelin‐associated glycoprotein
MAIs: myelin‐associated inhibitors
MAPK/ERK: mitogen‐activated protein kinase/Extracellular signal–regulated kinase
miRNAs: microRNAs
MSCs: mesenchymal stem cells
mTOR: mechanistic target of rapamycin
NgR: Nogo receptor
NgR3: Nogo receptor 3
Nogo: Neurite outgrowth inhibitor
NSCs: neural stem cells
NT‐3: neurotrophin‐3
OMgp: oligodendrocyte myelin glycoprotein
OPCs: oligodendrocyte progenitor cells
PAK1: p21‐activated kinase 1
PI3K/Akt: phosphoinositide 3‐kinase/protein kinase B
PirB: paired immunoglobulin‐like receptor B
PNS: peripheral nervous system
PTEN: phosphatase and tensin homolog
PTPσ: protein tyrosine phosphatase‐sigma
Rac1: Ras‐related C3 botulinum toxin substrate 1
RCT: randomized controlled trial
RhoA: Ras homolog family member A
ROCK: Rho‐associated coiled‐coil–containing protein kinase
scES: spinal cord epidural stimulation
SCI: spinal cord injury
shRNA: short hairpin RNA
SOCS3: suppressor of cytokine signaling 3
Sox11: SRY‐box transcription factor 11
STAT3: signal transducer and activator of transcription 3
TGFβ1/SOX9: transforming growth factor‐β1/SRY‐box transcription factor 9
TLR9: Toll‐like receptor 9
tSCS: transcutaneous spinal cord stimulation

## 1 Background

Spinal cord injury (SCI) refers to damage to the central nervous system (CNS) caused by traumatic or non‐traumatic events [1]. Patients often experience severe and persistent functional impairments, which significantly reduce their quality of life [2]. The incidence and prevalence of SCI have gradually increased, with reported incidence rates ranging from 8 to 83 cases per million and an estimated 250,000–500,000 new cases annually, affecting more than 2.7 million people worldwide [3, 4]. With global population aging, SCI in older adults has also risen [5]. According to the Global Burden of Disease Study, SCI imposes substantial direct and indirect costs due to acute care and long‐term rehabilitation, with particularly heavy impacts in low‐ and middle‐income countries [4, 6].

To date, the restoration of function following SCI remains a formidable challenge. Meaningful anatomical repair necessitates the re‐establishment of neural connections at the site of injury, with axonal regeneration being the key to this process [7]. However, axonal regrowth within the injured CNS is severely hindered by both extrinsic and intrinsic inhibitory factors. Extrinsic barriers include the stage‐dependent dual role of the glial scar, molecular inhibition mediated by myelin‐associated inhibitors (MAIs) and chondroitin sulfate proteoglycans (CSPGs), and a relative deficiency of neurotrophic and other growth‐promoting factors. Intrinsically, mature neurons exhibit diminished regenerative capacity. In response, strategies have been proposed to modulate glial scar dynamics, neutralize inhibitory molecules within the lesion microenvironment, reconstruct a permissive extracellular matrix (ECM), transplant stem or progenitor cells, apply biomaterial scaffolds, activate intrinsic neuronal growth programs, and employ neuromodulation.

Further elucidation of the mechanisms underlying regeneration failure after SCI and sustained advances in basic and translational research are crucial for improving patient prognosis and reducing healthcare and societal burdens. Compared with previous reviews that have primarily focused on individual inhibitory mechanisms or specific therapeutic categories, this review places particular emphasis on the translational implications of regenerative strategies. We first summarize the major barriers to axonal regeneration as interacting extrinsic microenvironmental constraints and intrinsic neuronal growth limitations. We then discuss representative therapeutic approaches in relation to their biological targets, injury‐stage considerations, available preclinical and clinical evidence, and unresolved translational challenges. This perspective is intended to provide a more structured overview of current SCI regenerative strategies and to inform the rational development of future combination therapies.

## 2 Axonal Regeneration Inhibitory Mechanisms

**FIGURE 1:**
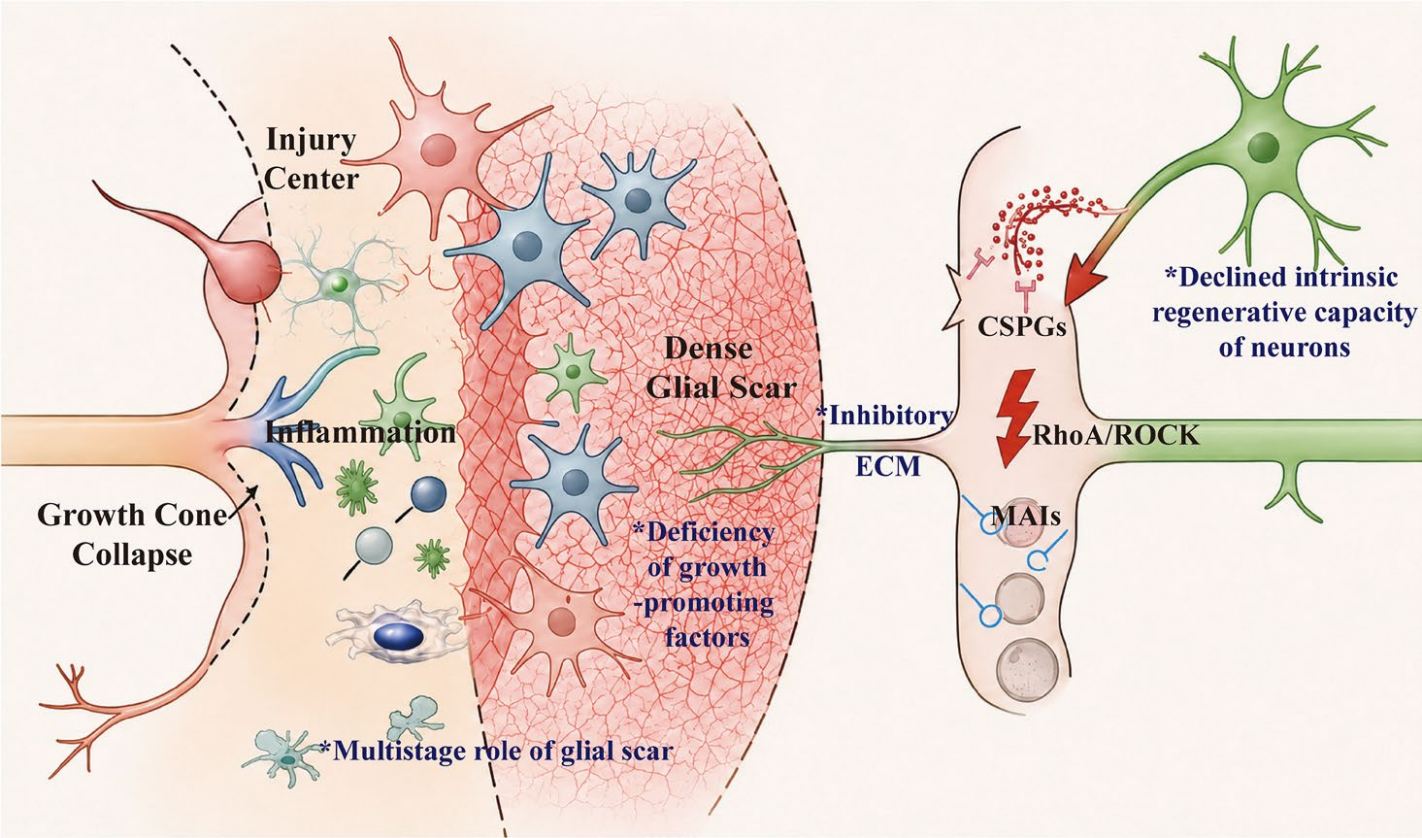
Mechanisms underlying the inhibition of axonal regeneration after SCI. After SCI, inflammation, glial activation, and lesion remodeling contribute to glial scar formation around the injury center. Although early glial scarring may help contain inflammation and protect spared tissue, chronic scar tissue forms a physical and biochemical barrier to axonal regrowth. Inhibitory ECM components, particularly CSPGs, together with MAIs, activate RhoA/ROCK‐related signaling, leading to growth cone collapse and suppression of axonal elongation. In parallel, insufficient growth‐promoting factors and reduced intrinsic regenerative capacity of mature neurons further limit regeneration. Together, these mechanisms create a non‐permissive environment for axonal repair. CSPGs, chondroitin sulfate proteoglycans; ECM, extracellular matrix; MAIs, myelin‐associated inhibitors; SCI, spinal cord injury.

Following SCI, axonal regeneration is constrained by chronic glial scarring, inhibitory ECM molecules, insufficient growth‐promoting factors, and the diminished intrinsic regenerative capacity of mature neurons (Figure 1). Importantly, these barriers should not be viewed as isolated mechanisms. Glial scarring provides both a cellular and molecular source of inhibitory cues, MAIs and CSPGs converge on shared downstream pathways such as RhoA/ROCK, and insufficient trophic support further limits the ability of injured neurons to respond to regenerative signals. Thus, regeneration failure reflects a multilayered interaction between the lesion microenvironment and neuron‐intrinsic growth programs.

### 2.1 The Biphasic Role of Glial Scarring

**FIGURE 2:**
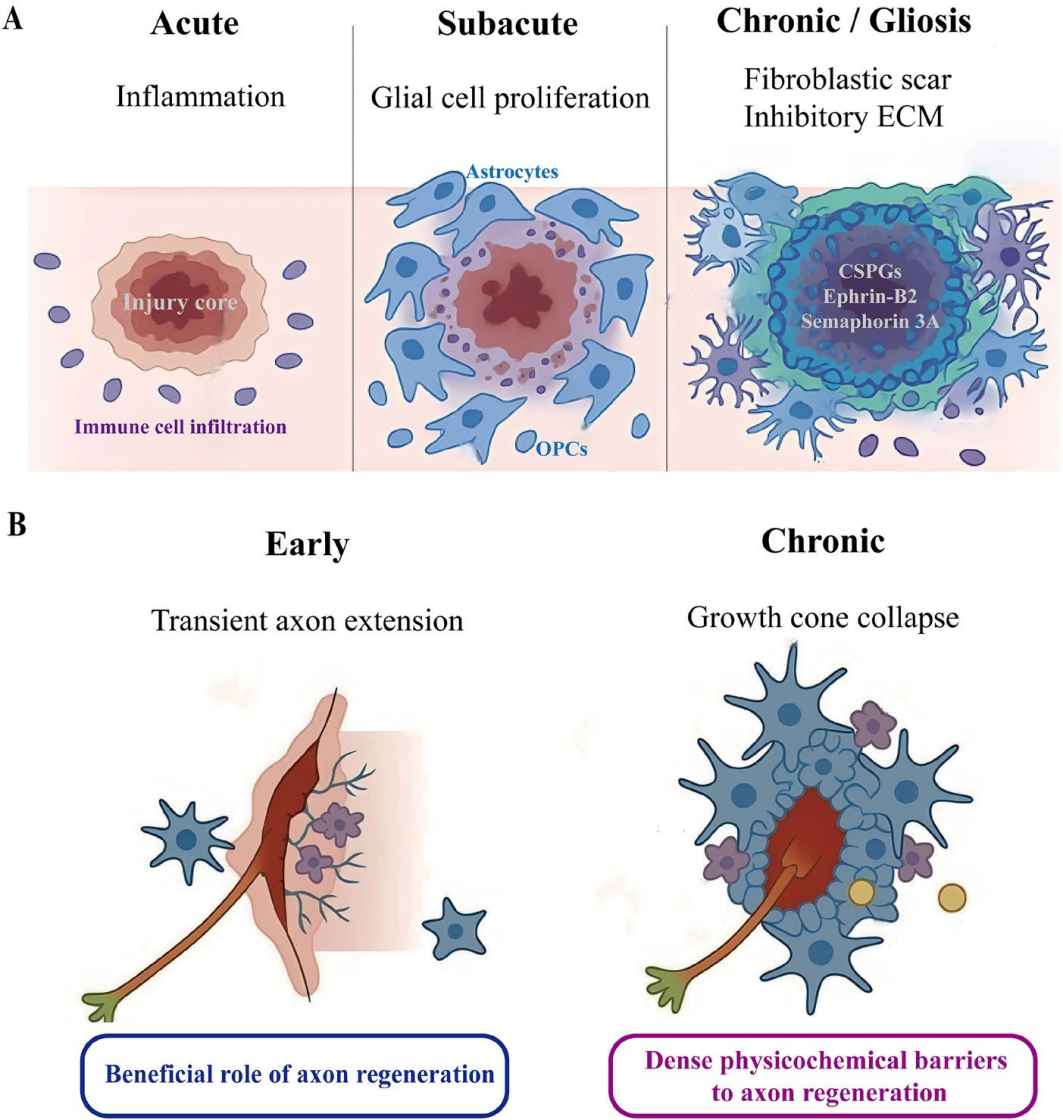
The biphasic role of glial scarring following SCI. (A) Schematic of post‐injury phases: Acute (intense inflammation), subacute (glial cell proliferation), and chronic (dense glial scar). (B) Axonal behavior in relation to scar development: Axons may transiently extend into the lesion during the acute phase, whereas chronic scar tissue forms a barrier that halts growth cones; ECM, extracellular matrix.

The failure of axonal regeneration after SCI has long been attributed to the inhibitory lesion environment, with the glial scar traditionally viewed as a major physical barrier [8]. The glial scar is a dense cellular border around the lesion core, composed primarily of reactive astrocytes, oligodendrocyte precursor cells, microglia, and infiltrating immune cells. These cells form a compact barrier that impedes axonal extension across the injury site [9, 10]. However, recent studies indicate that the glial scar is not merely a passive blockade; under certain conditions it may also support axonal regeneration within the CNS [11, 12, 13]. Ablation of the glial scar during the acute or subacute phase exacerbates lesion expansion and worsens functional recovery [13], whereas removal of scar tissue at later chronic stages can enhance axonal regrowth [14]. Thus, glial scarring is a temporally dynamic process with both protective and inhibitory roles that depend on injury stage and microenvironmental context [15]. During the acute to subacute phase (0–2 weeks post‐injury), activated astrocytes contain the lesion, limit inflammation, and help restore the blood–spinal cord barrier, thereby transiently protecting surrounding neural tissue [12]. Early‐reactive astrocytes also secrete neurotrophic factors such as brain‐derived neurotrophic factor (BDNF) and glial cell line‐derived neurotrophic factor (GDNF), supporting survival and function of residual neurons. By contrast, during the chronic phase (> 2 weeks post‐injury), the lesion border develops into a dense and restrictive matrix enriched in CSPGs and axon‐repelling molecules such as Ephrin‐B2 and Semaphorin 3A, forming a physical and biochemical barrier that severely restricts axonal regeneration [10]. Overall, the glial scar plays a complex, stage‐dependent role in SCI recovery. Early glial scarring facilitates tissue repair and neuroprotection, whereas chronic scars present formidable obstacles to axonal regeneration (Figure 2). From a therapeutic perspective, this biphasic pattern suggests that glial scar intervention should be stage‐sensitive rather than uniformly suppressive. Early strategies may need to preserve astrocyte‐mediated containment and neuroprotection, whereas later interventions may focus on reducing chronic scar‐associated molecular inhibition and improving axonal permissiveness.

### 2.2 Chemical Inhibition by Extracellular Growth‐Inhibitory Molecules

#### 2.2.1 Myelin‐Associated Inhibitors (MAIs)

After SCI, myelin structure and composition are altered, leading to activation of MAIs that suppress axonal regeneration via multiple signaling pathways. Major MAIs include neurite outgrowth inhibitor (Nogo), myelin‐associated glycoprotein (MAG), and oligodendrocyte myelin glycoprotein (OMgp) [16]. These molecules exert inhibitory effects by binding to neuronal receptors and triggering intracellular cascades that limit axonal outgrowth [17]. Nogo proteins, predominantly expressed by oligodendrocytes, include three isoforms (Nogo‐A, ‐B, and ‐C); Nogo‐A is the most prominent in the CNS and induces growth cone collapse and neurite inhibition [18]. MAG, localized within the myelin sheath, activates signaling pathways that lead to cytoskeletal retraction and suppression of neurite extension [19, 20]. OMgp, though less extensively studied, is also a potent inhibitor of neurite growth and induces growth cone collapse in adult dorsal root ganglion neurons [17]. These three MAIs share common receptors, including the Nogo receptor (NgR) and paired immunoglobulin‐like receptor B (PirB), which mediate growth inhibition [21, 22]. Ligand binding activates downstream signaling that disrupts the balance between small GTPases Rac1 and RhoA. Rac1 promotes cytoskeletal remodeling and growth cone extension via p21‐activated kinase 1 (PAK1), whereas RhoA induces growth cone collapse through Rho‐associated coiled‐coil–containing protein kinase (ROCK). MAI signaling enhances RhoA and suppresses Rac1 activity, resulting in growth cone collapse and suppression of axonal elongation [19, 23]. The convergence of Nogo‐A, MAG, and OMgp signaling on shared receptor complexes and downstream RhoA/ROCK activation indicates that MAI‐mediated inhibition is not a single‐ligand problem. Therapeutically, this supports strategies that target common receptors or downstream signaling nodes, although such approaches may still require combination with microenvironmental remodeling and intrinsic growth activation to achieve sustained regeneration.

#### 2.2.2 Chondroitin Sulfate Proteoglycans (CSPGs)

CSPGs are ECM molecules primarily secreted by reactive astrocytes, with additional contributions from macrophages and oligodendrocytes [24]. They are key inhibitory components of the glial scar. Following SCI, microglia promote CSPG production by astrocytes via the TGFβ1/SOX9 signaling pathway, and accumulated CSPGs reinforce scar structure and form a barrier that limits axonal passage through the lesion [24]. Beyond this physical blockade, CSPGs actively inhibit axonal regrowth by engaging specific receptors such as protein tyrosine phosphatase‐sigma (PTPσ), Nogo receptor 3 (NgR3), and leukocyte common antigen‐related receptor (LAR) [25, 26, 27, 28, 29]. Ligand–receptor binding activates RhoA/ROCK signaling, driving growth cone collapse and halting axon elongation [28, 30]. CSPG signaling can also upregulate other inhibitory molecules such as Ephrin‐B2 and Semaphorin 3A, further promoting growth cone collapse and exacerbating axonal inhibition [31, 32]. The combination of potent biochemical inhibition by CSPGs and the physical barrier of the glial scar creates a synergistic obstruction to axonal regeneration.

### 2.3 Deficiency of Growth‐Promoting Factors

Axonal regeneration failure after SCI results not only from multiple inhibitory molecules but also from a relative deficiency of neurotrophic and other growth‐promoting factors. During neural repair, growth factors regulate proliferation, differentiation, survival, migration, axonal guidance, and synaptogenesis [33]. They act through specific receptors to activate downstream pathways such as PI3K/Akt and MAPK/ERK, which regulate growth cone dynamics [34]. In the peripheral nervous system (PNS), abundant growth factors and a permissive ECM create an environment that supports robust regeneration [35, 36]. By contrast, the injured CNS accumulates inhibitory matrix molecules and lacks sufficient growth factors and pro‐regenerative cues [25], resulting in a severely impaired regenerative response. This imbalance between abundant inhibitory cues and insufficient trophic support helps explain why removing inhibitory molecules alone may be inadequate. Regenerative strategies may therefore need to combine disinhibition with spatially and temporally controlled delivery of neurotrophic and other growth‐promoting signals.

### 2.4 Decline in the Intrinsic Growth Capacity of Neurons

In the CNS, diminished intrinsic growth capacity of mature neurons is a key contributor to regeneration failure. Early work focused on extrinsic inhibitory molecules, but blocking these cues only modestly enhances axonal sprouting. Even on permissive substrates, adult neurons regenerate poorly [37], indicating that intrinsic factors are equally important. During development, CNS neurons progressively lose axonal growth potential: early‐stage neurons show robust regeneration, whereas this capacity declines with neuronal maturation. In adult neurons, selective axonal transport is impaired, hindering delivery of essential components to growth cones and compromising regeneration. Expression and function of regeneration‐associated receptors, such as integrins and growth factor receptors, also decline with neuronal maturation, further limiting responsiveness to external cues [38]. At the molecular level, intrinsic regenerative capacity is also regulated by growth‐associated signaling pathways. PI3K/Akt/mTOR [34] and JAK/STAT signaling contribute to neuronal growth responses, trophic signaling, and regeneration‐associated gene programs [39, 40, 41, 42, 43, 44, 45], whereas PTEN and SOCS3 negatively regulate these pathways [41, 42, 43, 44, 45]. Dysregulation of these intrinsic signaling programs may further limit the ability of injured neurons to respond to a more permissive extracellular environment. Additionally, injured neuronal cell bodies often exhibit atrophy, reduced metabolism, and impaired signal transduction, all of which further compromise intrinsic regenerative capacity [46, 47, 48]. These observations indicate that converting the lesion site into a more permissive environment may not be sufficient unless injured neurons are also reprogrammed toward a growth‐competent state. This provides the biological rationale for combining extrinsic microenvironmental modulation with approaches that activate intrinsic growth pathways.

### 2.5 Signaling Intersections Between Extrinsic Inhibition and Intrinsic Growth Programs

Although extrinsic microenvironmental barriers and intrinsic neuronal limitations are discussed separately above, they are mechanistically interconnected through several convergent signaling pathways. MAIs and CSPGs both activate receptor‐mediated inhibitory cascades that converge on RhoA/ROCK signaling, leading to cytoskeletal contraction, growth cone collapse, and suppression of axonal elongation [19, 21, 22, 23, 28, 30]. In parallel, growth‐promoting pathways such as PI3K/Akt/mTOR and JAK/STAT regulate neuronal responsiveness to trophic stimulation and regeneration‐associated gene programs [34, 39, 40, 41, 42, 43, 44, 45]. Negative regulators such as PTEN and SOCS3 further constrain these intrinsic responses by inhibiting PI3K/Akt/mTOR and cytokine‐related signaling, respectively [39, 40, 41, 42, 43, 44, 45]. Therefore, regeneration failure should not be viewed simply as the sum of extracellular inhibition and reduced intrinsic growth capacity. Rather, it reflects a network‐level imbalance in which convergent inhibitory signaling is combined with insufficient activation of pro‐regenerative pathways. This mechanistic convergence helps explain why targeting a single level of the regenerative barrier may be insufficient and provides a biological rationale for multi‐level repair strategies after SCI.

## 3 Therapeutic Strategies for Promoting Axonal Regeneration

**FIGURE 3:**
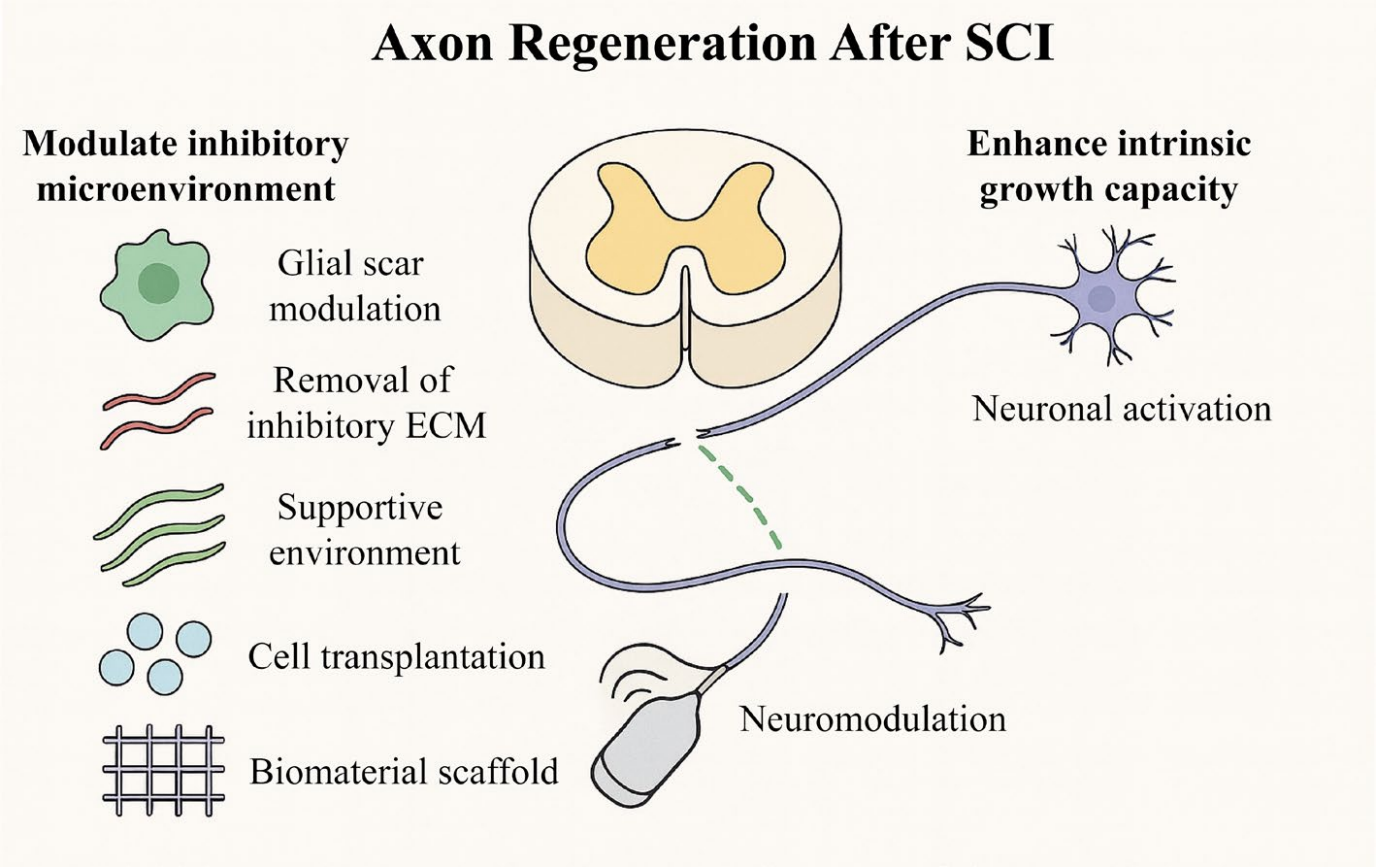
Therapeutic strategies for promoting axonal regeneration after SCI. Strategies to promote axonal regeneration after SCI target both extrinsic inhibitory barriers and intrinsic neuronal repair capacity. These include glial scar modulation, removal of inhibitory ECM components, reconstruction of a supportive environment, cell transplantation, biomaterial scaffolds, neuronal activation, and neuromodulation. These approaches may be combined according to injury stage and translational feasibility; ECM, extracellular matrix; SCI, spinal cord injury.

In response to multifactorial inhibition of axonal regeneration after SCI, diverse strategies have been developed to modulate the pathological microenvironment (Figure 3). These include modulation of glial scar responses, degradation of inhibitory ECM components, reconstruction of a permissive regenerative niche, cell transplantation, and biomaterial scaffolds, which mainly target extrinsic barriers. In parallel, strategies aimed at activating intrinsic neuronal growth programs and neuromodulatory interventions seek to enhance neuronal responsiveness, circuit excitability, and activity‐dependent plasticity. The following sections discuss these approaches in relation to their biological targets, available preclinical and clinical evidence, potential relevance to different injury stages, and major translational challenges. This organization is intended to highlight why single‐modality interventions have often shown limited effects and why rational combinations may be required for clinically meaningful repair. Table 1 summarizes these major strategies.

**TABLE 1:** Therapeutic strategies for promoting axonal regeneration after SCI.

Modulation of glial scar	Modulation of astrocyte activity Immune cell regulation Genetic approaches CSPG degradation
Elimination of inhibitory extracellular matrix	
Elimination of MAIs	Anti‐Nogo‐A antibody Receptor blockade Inhibition of downstream signaling pathways
Elimination of CSPGs	ChABC Receptor blockade Inhibition of downstream signaling pathways
Reconstruction of supportive regenerative environment	Reconstruction of growth‐supportive substrates Delivery of chemotactic growth factors
Cell transplantation	Direct intrathecal injection Combination therapy (gene editing; neurotrophic factors; biomaterial carriers)
Biomaterial scaffolds	Hydrogel Nanomaterial scaffold 3D bioprinted scaffold miRNA‐functionalized scaffold Multifunctional integrated scaffold Topological biomimetic scaffold
Activation of the intrinsic regenerative capacity of neurons	Activation of transcription factors Blockade of inhibitory signaling Restoration of energy metabolism Promotion of neuronal survival
Neuromodulation technologies	Transcutaneous spinal cord stimulation Spinal cord epidural stimulation/Spinal cord subdural stimulation Double‐target magnetic stimulation Extremely low frequency magnetic fields Photobiomodulation Optogenetic therapy

### 3.1 Modulation of Glial Scarring

Given the time‐dependent biphasic effects of glial scarring, current strategies emphasize modulation rather than simple removal. Key approaches include regulation of astrocyte activity, immunomodulation, and gene‐based modulation of scar‐associated cellular responses. Astrocytes proliferate excessively during the chronic phase and form a barrier that impedes regeneration. Fine‐tuning astrocytic responses is therefore critical. Signal transducer and activator of transcription 3 (STAT3) is a key regulator of astrocyte activation; inhibiting excessive STAT3 activity can reduce astrocyte proliferation and scar formation [49, 50]. A2‐type astrocytes have neurotrophic properties and may support axonal regeneration after SCI [51, 52]; therefore, promoting this phenotype could help reduce the inhibitory effects of chronic glial scarring [53, 54]. Immune cells such as microglia and macrophages infiltrate the injury site and secrete cytokines that influence scar formation. Targeting microglial receptors, suppressing inflammatory signaling, or inducing macrophage polarization toward the M2 phenotype can reduce detrimental scarring. For example, toll‐like receptor 9 (TLR9) antagonists modulate astrocyte–macrophage signaling and enhance M2 polarization [55], while Wnt4‐modified neural stem cells promote M2 polarization and tissue repair [56]. Gene therapy further allows precise regulation of scar‐related factors, such as the PI3K/Akt pathway [57], mTOR [58], or glial fibrillary acidic protein [59]. CSPGs are critical components of the glial scar; their direct degradation is discussed separately in Section 3.2.2.

Despite encouraging preclinical findings, scar modulation remains translationally challenging because it requires preservation of the early protective functions of glial scarring while reducing its chronic inhibitory effects. Excessive suppression during the acute phase may aggravate tissue damage, whereas insufficient modulation during the chronic phase may allow a persistent molecular and physical barrier to axonal regrowth. Future scar‐targeted strategies should therefore be guided by injury stage, astrocyte and microglial phenotypes, and the balance between protective scar containment and chronic inhibition.

### 3.2 Elimination of Inhibitory Extracellular Matrix

#### 3.2.1 Neutralizing Myelin‐Associated Inhibitors (MAIs)

Mitigating MAI‐mediated inhibition may facilitate axonal extension through the lesion site, particularly before scar maturation, and may thereby contribute to functional recovery. Early animal studies showed that removal of MAIs at or near the lesion facilitates axonal regeneration and extensive neurite outgrowth [60]. Anti‐Nogo‐A antibodies appear to be most effective when administered during the acute to subacute period, from within hours to approximately 14 days after injury, whereas delayed treatment produces limited regeneration [61]. A phase II trial of the humanized anti‐Nogo‐A antibody NG101 in acute cervical SCI did not improve upper‐limb motor function in the overall cohort, although potential benefit was suggested in patients with incomplete motor deficits [62].

Targeting common MAI receptors and downstream RhoA/ROCK signaling provides additional strategies. NgR1 and PirB act as shared receptors for the three principal MAIs [63]. NgR1 antagonists such as LOTUS may shift the lesion microenvironment from a non‐permissive to a more permissive state and thereby enhance regeneration [64, 65, 66]. The NgR1 antagonist AXER‐204 has shown a favorable safety profile and potential benefit in patients with chronic, incomplete SCI [67]. Inhibition of PirB, via antibodies or genetic modification, also attenuates MAI‐induced neurite inhibition [63]. Downregulation of RhoA or ROCK can counteract Nogo‐A‐mediated growth cone collapse [68, 69]. Clinically, the Rho inhibitor BA‐210 improved motor scores in a phase I/IIa trial [70], whereas the Rho inhibitor VX‐210 failed to significantly improve function in a phase IIb/III trial [71]. The failure of VX‐210 illustrates a broader translational challenge in SCI regenerative trials. Although RhoA/ROCK inhibition has strong mechanistic rationale and encouraging preclinical support, the phase IIb/III trial of VX‐210 did not meet its primary efficacy endpoint and was terminated after a predefined futility analysis. This negative result should not be attributed only to limited drug penetration with epidural delivery. It also highlights the difficulty of translating findings from standardized animal models to heterogeneous human SCI populations. Human SCI varies substantially in neurological level, injury completeness, lesion pathology, residual spared pathways, time from injury to treatment, spontaneous recovery potential, rehabilitation exposure, and responsiveness to outcome measures. Future trials of pathway‐targeted drugs should therefore incorporate more refined patient stratification, optimized lesion‐site delivery, and endpoints that are sensitive to biologically plausible treatment effects.

#### 3.2.2 Neutralizing the Inhibitory Effects of CSPGs

Given the strong inhibitory role of CSPGs, reducing CSPG‐mediated inhibition is an important therapeutic target. ChABC, a bacterial enzyme that degrades glycosaminoglycan side chains, significantly enhances axonal regeneration and collateral sprouting in animal models [72, 73, 74, 75]. However, its bacterial origin is associated with poor thermal stability and potential immune responses, which limit its clinical application. To improve its stability and local availability, biomaterial‐based delivery systems, including copolymers, thermosensitive hydrogels, and nanoparticles, have been explored for controlled ChABC release [76]. Gene‐based strategies using lentiviral or AAV vectors can also achieve stable and localized ChABC expression in the injured spinal cord [77, 78].

Targeting CSPG receptors, including PTPσ, NgR3, and LAR, and their downstream RhoA/ROCK signaling pathways offers complementary approaches. Small‐molecule inhibitors of PTPσ can enhance Trk receptor signaling and promote axonal elongation [79], whereas LAR‐targeting peptides can stimulate serotonergic axonal regeneration [80]. RhoA or ROCK inhibitors may further prevent CSPG‐induced growth cone collapse [75, 81]. Future studies should focus on improving the stability, specificity, and safety of CSPG‐targeted interventions. In addition, combinatorial strategies that simultaneously target multiple components of CSPG signaling may be required to achieve more robust regenerative effects.

### 3.3 Reconstructing a Supportive Regenerative Environment

Effective regeneration requires not only removal of inhibitory factors but also reconstruction of a supportive molecular microenvironment. Key elements include ECM remodeling and delivery of neurotrophic or growth‐promoting factors. ECM components such as laminin, fibronectin, and osteopontin provide permissive substrates that stimulate axonal regrowth [82]. Incorporating these substrates into hydrogels and nanomaterials can mimic native ECM and create a conducive microenvironment. Neurotrophic factors including BDNF, GDNF, hepatocyte growth factor (HGF), insulin‐like growth factor‐1 (IGF‐1), and neurotrophin‐3 (NT‐3) play indispensable roles in neuronal survival and axonal outgrowth [83]. Localized delivery via direct injection, biomaterial carriers, or gene transduction is widely used. For example, AAV‐mediated BDNF expression in human neural stem cell grafts promoted synaptic integration with host motor neurons [84], and NT‐3–releasing multichannel nanofiber scaffolds enhanced neuronal differentiation and synapse formation [85].

In clinical studies, subcutaneous administration of granulocyte‐colony stimulating factor improved neurological function in patients with SCI [86], but most clinical work still relies on simple injection approaches. Biomaterial‐mediated delivery and gene transduction show strong preclinical potential but lack clinical trials. Major translational hurdles include targeting precision and efficiency for biomaterial carriers and safety concerns for gene therapy, such as off‐target effects and viral immunogenicity. Addressing these barriers is essential for clinical implementation.

### 3.4 Cell Transplantation

Cell transplantation is a promising strategy for neural regeneration after SCI. Transplanted cells can repopulate the lesion core, receive afferent input from the host, and form functional synaptic connections with surviving neurons [87, 88]. Common cell sources include neural stem cells (NSCs), mesenchymal stem cells (MSCs), oligodendrocyte progenitor cells (OPCs), and Schwann cells [89, 90]. These cells can differentiate into neurons, oligodendrocytes, and astrocytes, thereby replacing lost neural cells, and they also exert paracrine effects by secreting cytokines and neurotrophic factors that modulate immunity, promote angiogenesis, and support endogenous repair [83, 91, 92]. To enhance efficacy and survival, transplantation is often combined with synergistic strategies. Genetic engineering can increase expression of survival‐related genes and optimize cell phenotypes [93, 94]. Co‐administration of neurotrophic factors, such as BDNF‐transfected MSCs, augments functional recovery [95]. Biomaterial‐based delivery systems, including thermosensitive hydrogels, can protect transplanted cells, modulate local inflammation, and promote sensorimotor recovery [96].

Early‐phase clinical trials indicate that transplantation of autologous bone marrow‐ or adipose‐derived MSCs and OPCs is feasible and generally safe, with observed neurological improvements in some SCI patients [97, 98, 99, 100, 101, 102, 103]. Trials of hiPSC‐derived neural stem/progenitor cells are ongoing [104]. Although MSC and OPC transplantation have progressed further clinically than many molecular or scaffold‐based strategies, current evidence should be interpreted primarily as safety and feasibility evidence rather than definitive proof of efficacy. MSC‐based studies have reported acceptable safety profiles in many settings, but controlled evidence for neurological improvement remains limited and heterogeneous. Similarly, OPC transplantation has shown encouraging safety and neurological‐function signals in early‐phase studies, but the available data are not yet sufficient to establish durable efficacy or optimal indications. Future studies should clarify whether cell transplantation is intended to replace lost cells, promote remyelination, provide paracrine support, modulate inflammation, or serve as a delivery platform for combined therapies.

### 3.5 Biomaterial Scaffolds

Biomaterial scaffolds offer structural and molecular support for SCI repair. Once implanted, they bridge lesion gaps, provide physical support for cells and tissues, and help reconstruct neural conduction pathways [105]. They can recapitulate key ECM features, offer permissive substrates for axonal extension [105], modulate immune responses, and suppress inflammation [106, 107]. Scaffolds also serve as delivery platforms for transplanted cells, growth factors, and regulatory molecules such as microRNAs (miRNAs) [108, 109]. Major scaffold types include hydrogels, nanomaterial‐based scaffolds, and three‐dimensional (3D) bioprinted constructs [83, 105, 109, 110, 111, 112, 113].

Animal studies have highlighted the promise of miRNA‐functionalized scaffolds [96, 114, 115], multifunctional integrated scaffolds [116], and topography‐inspired biomimetic scaffolds [117]. For example, miR‐219/miR‐338‐loaded fibrous hydrogels promote remyelination and functional recovery [115], while miRNA‐functionalized thermosensitive hydrogels can provide sustained release of therapeutic miRNAs, thereby modulating inflammation and apoptosis [96]. Multifunctional composite hydrogels can integrate ECM‐mimicking matrices, antioxidative nanoparticles, and MSC‐derived trophic factors to induce axonal regeneration [116]. Decellularized optic nerve and spinal cord matrices provide natural ECM and microtopographies that guide linear axonal growth [108, 117]. However, structural bridging alone does not ensure functional reconnection. Clinically effective scaffolds must also support vascularization, host–graft integration, controlled biodegradation, and compatibility with rehabilitation or neuromodulation. Despite encouraging preclinical progress, translation remains limited by insufficient large‐animal validation, incomplete long‐term biosafety data, and challenges in scalable manufacturing and quality control. Future studies should therefore strengthen safety and efficacy testing in clinically relevant models and optimize reproducible scaffold production.

### 3.6 Activation of the Intrinsic Regenerative Capacity of Neurons

Enhancing intrinsic neuronal regenerative capacity is a major strategy for promoting repair after SCI. Building on the intrinsic mechanisms described above, current approaches include activation of pro‐regenerative signaling pathways and transcriptional regulators, suppression of intrinsic inhibitory molecules, restoration of energy metabolism, and promotion of neuronal survival. Activation of mTOR‐related signaling, Kruppel‐like factors (KLFs), STAT3, B‐RAF, and Sox11 has been reported to upregulate regeneration‐associated programs and enhance neuronal responsiveness to growth cues [39, 40, 118, 119, 120, 121]. In parallel, inhibition of intrinsic suppressors such as PTEN and SOCS3 can promote axonal regrowth by enhancing PI3K/Akt/mTOR‐ and STAT3‐related signaling [41, 42, 43, 44, 45]. These strategies support the concept that mature CNS neurons may regain part of their growth competence when intrinsic inhibitory programs are relieved. Restoration of neuronal energy metabolism is another important approach. Bioenergetic compounds such as creatine may improve axonal regeneration [122], while mitochondria are key regulators of neuronal survival and regenerative responses [123]. Photobiomodulation and zinc supplementation have been reported to improve mitochondrial bioenergetics through AMPK‐related mechanisms, increase glucose uptake and mitochondrial biogenesis, and reduce oxidative stress and apoptosis [124, 125]. Other approaches, such as ginsenoside Rb1 treatment and mitochondrial transplantation, may further support mitochondrial homeostasis and repair after SCI [126, 127]. In addition, strategies that promote neuronal survival or reduce neuronal atrophy, such as lithium salts or reduction of synuclein accumulation, may support long‐distance regeneration and neurological recovery [47, 128]. Despite their biological promise, intrinsic growth activation strategies require cautious translational interpretation. PI3K/Akt/mTOR and JAK/STAT pathways, together with transcriptional reprogramming approaches, influence broad cellular processes, including survival, proliferation, inflammation, and tumor‐related signaling. Therefore, future approaches should aim for cell‐type‐specific, temporally controlled, and preferably reversible modulation, rather than nonspecific or prolonged activation.

### 3.7 Neuromodulation Technologies

Neuromodulation uses electrical, magnetic, ultrasonic, or optical modalities to modulate neural circuits and re‐engage ascending and descending pathways. Electrical stimulation approaches include transcutaneous spinal cord stimulation (tSCS) and implantable spinal cord stimulation. tSCS delivers current through cutaneous electrodes placed over the vertebral column and has been applied clinically with supportive multicenter clinical evidence [129]. Implantable spinal cord stimulation, delivered via epidural or subdural electrodes, modulates spinal network excitability and can create a permissive milieu for regeneration [130]. Spinal cord epidural stimulation (scES), combined with activity‐based rehabilitation, has been shown to promote functional recovery in individuals with SCI [131]. Subdural electrical field therapy has demonstrated safety and efficacy in thoracic SCI rat models [132]. Conductive biomaterial scaffolds integrated with electrical stimulation can also guide oriented axonal extension across lesions [133]. Magnetic stimulation approaches such as double‐target magnetic stimulation (DTMS) synchronously target the motor cortex and spinal circuits distal to the lesion, forming a bidirectional neuromodulatory loop that outperforms single‐target stimulation in restoring function and tissue architecture in SCI rats [134]. Mechanistically, DTMS upregulates growth‐associated protein 43 (GAP‐43) and mitigates oligodendrocyte apoptosis and oxidative stress, thereby promoting axonal regeneration and remyelination with consequent functional improvement [134]. Extremely low‐frequency magnetic fields (ELF‐MF) enhance cellular proliferation and differentiation and exert neuroprotective, anti‐inflammatory, and antioxidative effects [135, 136, 137]. Ultrasound stimulation, particularly low‐intensity pulsed or focused ultrasound, facilitates neural stem cell proliferation and differentiation, upregulates neurotrophic factors and regeneration‐associated genes, and can activate spinal circuits and alleviate spasticity [138, 139, 140, 141]. Photobiomodulation with low‐level laser irradiation modulates immune responses, attenuates inflammation, and reduces CSPG accumulation that impedes axonal growth [142, 143, 144, 145]. Optogenetic interventions combine optical techniques with genetic engineering to selectively activate or inhibit defined neuronal populations using light‐sensitive opsins, thereby promoting circuit remodeling, enhancing regeneration, and improving functional outcomes after SCI [146, 147, 148, 149, 150]. Among these neuromodulatory approaches, tSCS and scES are currently the most clinically advanced, whereas magnetic stimulation, ultrasound stimulation, and optogenetic strategies remain mainly experimental. Further protocol optimization and biomarker‐based studies are needed to clarify their mechanisms and translational value.

Taken together, the strategies discussed above should not be regarded as equally mature or equally close to clinical application. Overall, the therapeutic strategies reviewed in this section differ substantially in their evidence maturity and translational readiness. ChABC‐mediated CSPG degradation, hydrogels, miRNA‐functionalized scaffolds, intrinsic growth activation, gene‐based modulation, ultrasound stimulation, magnetic stimulation, and optogenetic approaches have strong mechanistic rationale and varying degrees of preclinical support, but they remain largely at the preclinical or early translational stage. Anti‐Nogo‐A therapy, NgR1‐targeted inhibition, RhoA/ROCK inhibition, MSC transplantation, OPC transplantation, and selected growth factor‐based strategies have entered clinical investigation, but their efficacy remains uncertain and heterogeneous across studies. In comparison, neuromodulatory approaches, particularly tSCS and scES combined with structured rehabilitation, have accumulated relatively more clinical evidence for functional improvement. However, functional improvement should not be directly equated with anatomical axonal regeneration, as it may reflect enhanced excitability of spared circuits, synaptic plasticity, or improved responsiveness to rehabilitation. Therefore, the translational potential of each strategy should be interpreted not only according to mechanistic plausibility or animal‐model findings, but also in relation to the maturity of the available evidence, therapeutic timing, delivery feasibility, safety, patient selection, endpoint sensitivity, and their potential to be incorporated into biologically rational combination therapies. Table 2 summarizes the current evidence, clinical status, and translational challenges of representative therapeutic strategies for SCI regeneration.

**TABLE 2:** Current clinical evidence and translational challenges of representative therapeutic strategies for SCI regeneration.

Therapeutic strategy	Representative clinical evidence/stage	Evidence interpretation	Major translational challenges
Anti‐Nogo‐A antibody	Phase II/IIb clinical trial	Strong mechanistic and preclinical rationale; Early clinical evidence suggests potential benefit, particularly in selected incomplete cervical SCI subgroups, but overall efficacy requires further confirmation	Patient selection; Treatment window; Confirmation of efficacy in larger controlled trials; Identification of responders
NgR1 antagonist	First‐in‐human randomized clinical trial	Early clinical evidence mainly supports safety, pharmacokinetics, and target engagement, efficacy remains exploratory	Optimal dose and repeated administration schedule; Patient stratification; Confirmation of functional efficacy
RhoA/ROCK inhibitor	Phase IIb/III trial completed; Negative/equivocal clinical result	Strong preclinical rationale, but VX‐210 failed to meet the primary efficacy endpoint and was terminated after futility analysis, this represents an important translational gap	Lesion‐site drug delivery; Insufficient local drug exposure; Patient heterogeneity; Endpoint sensitivity; Development of more efficient delivery systems
Growth factor‐based therapy	Representative Phase III evidence for G‐CSF; Other neurotrophic factor delivery approaches remain mainly preclinical or early clinical	Some clinical evidence exists for systemic growth factor‐related therapy, but biomaterial‐mediated delivery and gene‐transfer approaches are not yet clinically mature	Short half‐life; Limited targeting precision; Current clinical use mainly relies on systemic/subcutaneous administration; Technical and safety challenges for biomaterial carriers and gene transfer
Cell transplantation	MSCs: multiple early clinical studies; OPCs: Phase I/IIa clinical trial	Clinical studies generally support feasibility and acceptable safety, but robust and durable efficacy remains uncertain, neurological improvements are heterogeneous across studies	Cell survival and integration; Immune response; Cell‐source heterogeneity; Optimal dose and route; Need for large‐scale randomized controlled trials
Biomaterial scaffolds/hydrogels	Mainly preclinical; Limited clinical translation	Strong preclinical support as structural bridges and delivery platforms, but clinical evidence remains insufficient	Long‐term biosafety; Biodegradation control; Manufacturing standardization; Scalability; Integration with host tissue
miRNA‐functionalized scaffolds	Preclinical stage	Mechanistically promising for modulating inflammation, apoptosis, remyelination, and local microenvironment, but no mature clinical evidence	Delivery specificity; Off‐target effects; Controlled release; Long‐term safety; Clinical‐grade manufacturing
Transcutaneous spinal cord stimulation	Pivotal multicenter clinical trial; Relatively stronger clinical evidence for functional improvement	tSCS combined with structured rehabilitation has accumulated comparatively stronger clinical evidence for improving hand and arm function, but functional gains should not be interpreted as direct evidence of anatomical axonal regeneration	Standardized stimulation parameters; Patient selection; Durability of effect; Long‐term safety; Distinction between functional recovery and true axonal regeneration
Spinal cord epidural stimulation	Human clinical studies and early trials; Evidence mainly from small cohorts and selected patient groups	scES has shown functional improvements in selected patients, especially when combined with intensive rehabilitation, but larger controlled trials are still needed	Surgical invasiveness; Stimulation parameter optimization; Patient selection; Placebo/control design; Validation through larger RCTs
Intrinsic growth activation/gene‐based modulation	Mainly preclinical	Strong mechanistic rationale, especially for PTEN/SOCS3/mTOR‐related pathways, but clinical translation remains limited by safety and delivery concerns	Cell‐type specificity; Temporal control; Off‐target effects; Tumor‐related signaling risk; Safe and reversible delivery systems
Ultrasound, magnetic stimulation, and optogenetic approaches	Mainly preclinical or early experimental clinical exploration	Promising experimental neuromodulatory approaches, but evidence for SCI axonal regeneration in humans remains limited	Mechanistic uncertainty; Stimulation targeting; Protocol standardization; Safety; Need for rigorous translational studies

## 4 Summary and Outlook

Failure of CNS axonal regeneration remains a central barrier to functional recovery after SCI. This review summarized the multifactorial mechanisms that restrict axonal regrowth and the corresponding therapeutic strategies developed to overcome them. Degradation of CSPGs and neutralization of MAIs can alleviate local chemical inhibition, but these approaches may be insufficient to establish a sustained pro‐regenerative environment. Cell transplantation provides potential cellular and paracrine support, but its translation is limited by cell survival, graft integration, immune responses, and long‐term efficacy. Biomaterial scaffolds and neuromodulation may provide structural or functional support for repair, but they also face challenges related to biosafety, scalability, protocol optimization, and mechanistic uncertainty. These limitations suggest that single‐modality interventions are unlikely to fully address the complex biological demands of SCI repair. Rational combination strategies may therefore be necessary.

A key translational implication is that combination therapies should be considered according to the biological phase of SCI rather than applied as uniform treatment packages. In the acute to subacute stage, the main therapeutic priorities may include tissue protection, inflammation control, preservation of beneficial astrocytic responses, and early reduction of MAI‐mediated growth inhibition. At this stage, interventions should avoid disrupting the protective functions of early glial scar formation while limiting early molecular inhibition. In the subacute to early chronic stage, when inhibitory ECM components accumulate and trophic support remains insufficient, strategies such as ECM remodeling, controlled growth factor delivery, and biomaterial‐supported delivery of cells or therapeutic molecules may be more relevant. In the chronic stage, scar maturation, reduced intrinsic neuronal growth capacity, and limited circuit plasticity become major barriers. Therefore, combinations involving scar remodeling, intrinsic growth activation, neuromodulation, and intensive rehabilitation may be required to engage spared circuits and promote functional plasticity. This phase‐informed view is not intended to serve as a rigid treatment algorithm, but rather as a biologically grounded framework for considering when and why specific therapeutic combinations may be more appropriate. Table 3 provides a phase‐informed summary of translational considerations for combination therapeutic strategies after SCI.

**TABLE 3:** Phase‐informed translational considerations for combinatorial strategies after SCI.

Injury stage	Dominant biological barriers	Potential therapeutic focus	Biologically plausible combinations	Key translational concerns
Acute	Inflammation, tissue loss, early MAI signaling, unstable lesion environment	Neuroprotection, inflammation control, preservation of protective scar functions	Anti‐inflammatory modulation + early MAI inhibition + rehabilitation preparation	Safety, timing, avoiding disruption of protective scar
Subacute	Evolving glial scar, CSPG accumulation, insufficient trophic support	ECM remodeling, trophic support, scaffold/cell delivery	ChABC + growth factors + biomaterial scaffolds; cell transplantation + trophic support	Delivery stability, immune response, lesion heterogeneity
Chronic	Mature scar, reduced plasticity, low intrinsic growth capacity	Scar remodeling, intrinsic growth activation, circuit engagement	ChABC/scar modulation + intrinsic activation + neuromodulation + rehabilitation	Long‐term safety, patient selection, functional endpoints

Despite this rationale, the clinical translation of combination therapies remains challenging. At the treatment‐design level, major obstacles include suboptimal timing among therapeutic components, conflicts in delivery routes, uncertainties in biocompatibility and coordination across modalities, incomplete understanding of mechanistic complementarity, potential risk additivity, and the need for robust long‐term safety and efficacy data. At the preclinical‐to‐clinical translation level, most experimental studies still rely heavily on rodent models, in which injury severity, lesion level, treatment timing, and post‐injury care can be highly standardized. In contrast, human SCI is much more heterogeneous in neurological level, injury completeness, lesion pathology, spared descending or propriospinal pathways, disease stage, comorbidities, rehabilitation exposure, and potential for spontaneous recovery. These differences may partly explain why interventions with strong mechanistic rationale and encouraging animal data do not always produce consistent clinical benefits. Although several phase I/II studies have supported the safety and potential efficacy of agents targeting extrinsic inhibitory signals, cell transplantation, neurotrophic factors, and selected neuromodulatory techniques, high‐quality randomized controlled trials with long‐term follow‐up remain limited, and standardized treatment parameters are still lacking.

Future research should therefore focus on several priorities. First, large‐animal models that better approximate human spinal cord anatomy and injury complexity are needed to improve translational relevance. Second, the biological complementarity of combination therapies should be investigated more systematically, particularly with regard to injury stage, therapeutic sequence, and potential risk interactions. Third, more efficient and controllable delivery systems, such as smart controlled‐release biomaterials and targeted viral vectors, should be developed with careful attention to safety and reversibility. Fourth, preclinical studies should be more closely linked to early clinical trial design through clearer translational criteria, standardized outcome measures, and clinically meaningful endpoints. Finally, multicenter randomized controlled trials with adequate sample sizes and long‐term follow‐up are needed to evaluate safety, efficacy, durability, and applicability across different SCI subtypes and patient populations.

In conclusion, functional reconstruction after SCI will likely require coordinated progress in mechanistic research, therapeutic technology, and clinical trial design. Future strategies should move beyond isolated interventions toward phase‐informed, biologically rational, and clinically testable combinations. Careful validation of these approaches will be essential to determine whether they can produce safe, durable, and meaningful functional recovery after SCI.

## Author Contributions

Jiaxin Gao: writing – original draft; Tian Li: writing – review and editing; Qinping Su: writing – review and editing; Cai Jiang: supervision; Zhonghua Lin: supervision, funding acquisition.

## Funding

This work was supported by the key Discipline Construction Program of Traditional Chinese Medicine in Fujian Province (Project Number: 0060380308) and the 2022 General Project Plan of Fujian Medical University's Qihang Fund (Project Number: 2022QH1328).

## Conflicts of Interest

The authors declare no conflicts of interest.

## Data Availability

The data that support the findings of this study are available from the corresponding author upon reasonable request.
